# An interdisciplinary intervention to prevent falls in community-dwelling elderly persons: protocol of a cluster-randomized trial [PreFalls]

**DOI:** 10.1186/1471-2318-11-7

**Published:** 2011-02-17

**Authors:** Wolfgang A Blank, Ellen Freiberger, Monika Siegrist, Peter Landendoerfer, Klaus Linde, Tibor Schuster, Klaus Pfeifer, Antonius Schneider, Martin Halle

**Affiliations:** 1 Institute of General Practice, Technische Universitaet Muenchen, Muenchen, Germany; 2 Institute of Sports Science and Sports, University Nuernberg Erlangen, Germany; 3 Department of Prevention, Rehabilitation and Sports Medicine, Technische Universitaet Muenchen, Germany; 4 Institute for Medical Statistics and Epidemiology, Technische Universitaet Muenchen, Germany

## Abstract

**Background:**

Prevention of falls in the elderly is a public health target in many countries around the world. While a large number of trials have investigated the effectiveness of fall prevention programs, few focussed on interventions embedded in the general practice setting and its related network. In the Prevent Falls (PreFalls) trial we aim to investigate the effectiveness of a pre-tested multi-modal intervention compared to usual care in this setting.

**Methods/Design:**

PreFalls is a controlled multicenter prospective study with cluster-randomized allocation of about 40 general practices to an experimental or a control group. We aim to include 382 community dwelling persons aged 65 and older with an increased risk of falling. All participating general practitioners are trained to systematically assess the risk of falls using a set of validated tests. Patients from intervention practices are invited to participate in a 16-weeks exercise program with focus on fall prevention delivered by specifically trained local physiotherapists. Patients from practices allocated to the control group receive usual care. Main outcome measure is the number of falls per individual in the first 12 months (analysis by negative binomial regression). Secondary outcomes include falls in the second year, the proportion of participants falling in the first and the second year, falls associated with injury, risk of falls, fear of falling, physical activity and quality of life.

**Discussion:**

Reducing falls in the elderly remains a major challenge. We believe that with its strong focus on a both systematic and realistic fall prevention strategy adapted to primary care setting PreFalls will be a valuable addition to the scientific literature in the field.

**Trial registration:**

NCT01032252

## Background

Among elderly people falls are a major health problem associated with significant morbidity and economic burden. Based on the available data it is estimated that about 30% to 40% of community-dwelling older persons experience at least one fall per year with a high proportion falling more often than once [1-3]. These figures might be even underestimates. As patients and physicians consider falling and gait disorders as rather normal in older persons it is likely that there is a considerable underreporting of falls [4,5]. Major problems associated with falls include injury and fracture, reduced mobility, fear of falling, depression, admission to long-term care, decreased quality of life and death [6,7]. In Germany, about 125.000 persons per year suffer of mostly fall-induced hip fractures causing treatment costs of approximately 1.5 - 2 billion Euro [8,9]. Also, falls have a major role in about 26% of admissions to nursing homes leading also to major long term costs [10-12]. Lasting functional impairment occurs in 50% of fall patients with a hip fracture and 10% to 20% need permanent care [13]. Furthermore, psychological consequences such as fear of falling induce a negative vicious circle due to restriction of physical activities, thus decreasing quality of life and accelerating the process of functional decline of the aging process [14,15]. Given the demographic changes in many countries the prevalence of fall-related problems is likely to further increase in future [1,9].

A large number of clinical trials have investigated strategies to prevent falls or injurious falls. These studies have been summarized in several systematic reviews [3,10,16,17]. The recently published Cochrane review by Gillespie et al. focussing on fall prevention among older people living in the community includes 111 randomised controlled trials with a total of 55,303 participants [2]. There is some evidence for multiple-component group exercises, Tai Chi and individually prescribed multiple-component home-base exercises to reduce risk of falling and rate of falls [2,18]. Assessment and multifactorial intervention reduces rate of falls, but not significantly the general risk of falling [2,19-21]. Home safety interventions do not considerably reduce falls, but were effective in people with severe visual impairment and in others at higher risk of falling [2,22]. Also, anti-slip shoe device reduced rate of falls in icy conditions [2,23]. Furthermore, a prescribing modification programme for primary care physicians significantly reduces risk of falling [2,24] and gradual withdrawal of psychotropic medication reduces rate of falls, but not significantly risk of falling [2,25].

The systematic reviews [2,16] raised some methodological concerns: "Falling" was not always clearly defined and many trials were not powered adequately to detect clinically relevant effects. Many studies reported either rate of falls or the number of fallers although the Prevention of Falls Network Europe (ProFaNE) recommends to report both [26]. The duration of follow up was criticized to last only 12 months or shorter. Furthermore, one review demands that future trials should also include measurements of quality of life [16].

Several current guidelines [5,27-29] recommend multifactorial risk assessment of falls and interventions tailored to an individual's risk factor profile as a primary treatment strategy. However, implementing such strategies is far from easy. According to a qualitative study elderly persons regard fall prevention advices as generally useful but not personally relevant [30]. The likelihood that they take up fall prevention activities depends strongly on the setting and the characteristics of the intervention offered [31]. Older people view their general practitioners as a very important source of health-related information and value their advice [32,33]. Therefore, general practitioners are in an ideal position to identify persons at risk, and to recruit and motivate them to adhere to fall prevention programs [34]. While a large number of trials have investigated the effectiveness of fall prevention programs few focussed on interventions embedded in the general practice setting and its related network [16].

### Objectives

The primary aim of PreFalls is to investigate whether the implementation of a multimodal intervention in the German primary care setting (general practitioners), consisting of 16 weeks of group exercise in combination with an individualized home training program will significantly reduce number of falls per individual and risk of falling in community-dwelling elderly with high risk of falls compared to usual care. The secondary aim is to explore effects on the incidence and frequency of fall-related injuries, on balance, strength and mobility, fear of falling and quality of life. For this study a fall is defined as "an unexpected event in which the participant comes to rest on the ground, floor or at a lower level" [26].

## Methods/Design

### Study design and randomization

PreFalls is a controlled multicenter prospective study with cluster random allocation of participating general practices to an experimental or a control group. The study is performed in two regions (greater Munich and Nuremberg areas) in Bavaria, southern Germany. Two randomisation lists are computer generated by a statistician otherwise not involved in the study. The lists are stored by the two regional study coordinators. After the definitive inclusion of a general practice into the study, the coordinator of the respective region calls the coordinator of the other region who, after registering the practice, hands out the allocation information.

### Ethics

The study protocol was approved by the ethics board of the faculty of medicine of the Technische Universitaet Muenchen. The study design takes into account the principles set out in the Helsinki declaration (Seoul, 2008). All patients have to sign informed consent forms.

### Participating practices

General practitioners interested in participating in the trial are recruited from local peer groups (quality circles), from networks affiliated to the Institute for Family Medicine, Technische Universitaet Muenchen and the Institute of Sports Science and Sports, University Nuremberg/Erlangen as well as by advertising in medical journals. Participating physicians have to be licensed general practitioners working with elderly persons in a community dwelling setting. Physicians and one staff member (medical secretary, receptionist, assistant) of each participating practice in both experimental and control group are trained in workshops lasting 3.5 hours. The workshop introduces goals, methods and logistics of the study, provides background knowledge on falls (definitions, epidemiology and recommended guidelines) and includes a practical training part on the methods for fall risk assessment. For the attendance of the workshop physicians gain Continuing Medical Education (CME) points.

### Participating patients

The target population of the study are community-dwelling persons aged 65 years and older with a high risk of falls. To be included at least one of the following criteria has to be met (see section measurements for a detailed description of the tests): fall within the last 12 months, fear for falling [35], chair-stand-ups >10 sec. [36], Timed-up-and-go-Test >10 sec. [37,38], balance impairments measured by the three balance tests of the Guralnik short physical performance battery [36] or self-reported subjective balance deficits. Persons not living independently and patients suffering from physical or mental restrictions that do not allow participation in an exercise program or the assessment of risk of falling are excluded. Each participating practice is asked to recruit 10 to 15 patients. In case of problems with recruiting the regional coordinating team supports recruitment activities.

### Intervention

Physicians taking part in the study are asked to name a certified local physiotherapist or an university trained sport scientist interested to act as exercise instructor (trainer) for the fall prevention program. If the general practitioner cannot name a trainer the coordination team will identify a qualified person. If the practice is randomised to intervention program the study centre invites the trainer to a special course lasting 8 hours. The course covers the standardised evaluated intervention program [39-41] as well as other study-related issues such as definition of falls and assessment procedures to enable trainers to answer upcoming questions of the participants.

The intervention investigated in PreFalls is a 16 week program with focus on fall prevention. It is based on a program which has been successfully tested in a previous trial [40] but expanded by a fear of falling module. Briefly it contains a progressive strength and flexibility training, challenging balance, gait and motor coordination training and progressive endurance training (Table 1). Sessions take place once a week and last 60 minutes each. The multi-modal intervention focuses on the improvement of motor control with several components relevant for falls prevention: balance, gait, motor coordination in daily routine activities, and strength training. The intervention incorporates, both, group and home-based exercises as well as physical activity recommendations and educational components for fall prevention. The strength training includes progressive body weight-bearing exercise and functional power training. Balance training contains, for example, standing balance, dynamic weight transfers, stepping strategies. Gait training included walking with change of pace and direction. For daily activity performance, exercise included motor coordination under time pressure and sensory awareness. The exercise intervention also includes strategies for getting up from the floor. Given the importance of fear of falling, components of the "Matter of Balance" program [42] have been added to the intervention program to address not only physical but also psychological risk factors for falls. This cognitive behavioural program aims to reduce fear of falling by increasing self-efficacy. The evidence-based components of restructuring misconception of fall risk, attitudes about falls, as well as individual goal setting of changed behaviours for fall risk reduction are addressed [40,41].

**Table 1:** Intervention Details

Component	Sessions	Percentage
Strength Training	5	11%
Training of Perception & Components of Motor Coordination	10	21%
Flexibility Training	6	13%
Endurance Training/Gait & Balance Training	5	30%
Fall Risk Education	5	11%
Home Programm	2	4%
Fear Avoidance	3	7%

### Measurements

Table 2 provides an overview of the type and timing of measurements in PreFalls. Assessment visits take place at baseline (inclusion = month 0), at 4, 12 and 24 months. Falls are monitored by patients over the full study period with a daily fall calendar.

**Table 2:** Study schedule

Month	0	4	12	24
Sociodemographic characteristics	X			
Weight, height	X	X	X	X
Morbidity, medication	X	X	X	X
				
Risk of Fall assessment (Timed "Up and Go" Test, Chair Rising test, Romberg-Test, Tandem and Semi-Tandem Stand)	X	X	X	X
				
EuroQol Quality of Life questionnaire	X	X	X	X
Falls Efficacy Scale (FES-I) questionnaire	X	X	X	X
PAR questionnaire for physical activity level	X	X	X	X
	XXXXXXXXXXXXXXXXXXXXX			
Fall diary				
				
Fall prevention program (intervention group only)	XXXXXXXXX			

For the full study period of 24 months participants are asked to fill in a fall diary. For each day they mark whether they fell or tripped. The monthly calendar is sent to the study coordinators during the first week of the following month in a prepaid envelope. When a fall occurs, detailed information is obtained through structured telephone interviews (see Table 3). If the diary is not received within 10 days after the end of the documented month the participant is contacted by phone. In case that during follow-up some patients stop to fill in the diary they will be phoned to ask for basic information on falls (number of falls, injuries and consequences).

**Table 3:** Questions asked in the telephone interview after documentation of a fall in the diary

How often nearly fallen in the previous month?
How often fallen in the previous month?
Where did the last fall happen?
Getting up without help?
Time of fall?
Reason for falling?
Injuries?
Doctor consultation?
Which doctor consulted?
Admission to hospital?
Kind of shoes at time of fall?
Used glasses at time of fall?

At each study visit the risk of fall is assessed. In the "timed up and go test" (TUG) subjects are asked to stand up from a standard chair with a seat height of between 40 and 50 cm, walk a distance of three meters at a normal pace, turn, walk back to the chair and sit down. Measurement of time (in seconds) begins at the word "go" and ends when the subject's back touching the backrest of the chair, with a shorter time taken indicating better balance ability and mobility [38,43]. The "chair stand ups-test" measures the time it takes to complete five rapid chair rise cycles (sit to stand to sit) and is an indicator of the functional strength of the lower extremity and the dynamic balance capability [44]. The static balance measurement is taken from the "Short Physical Performance Battery" (SPPB) by Guralnik et al. and consists of three measurements (side by side position, semi-tandem, and tandem position) [36].

Fear of Falling is assessed with the German version of the Falls Efficacy Scale International (FES-I) [35], physical activity by the 7-Day Physical Active Recall (PAR) [45], and quality of life with the EuroQol [46]. Furthermore, patients are asked to rate their overall health status using the visual analogue scale (range 0-100, higher scores indicating better health). All tests and questionnaires used in the study have demonstrated good validity and reliability and are well known in this field.

### Sample size justification

Sample size calculation was performed using IcebergSim software (version: Beta 3.06, Bergel 2005-2006), which allows considering intracluster correlation, which is a serious issue in cluster-randomized trials.

Calculations are based on assumptions regarding the proportion of individuals with at least one fall during a 12-month follow up period. Since expected numbers of falls per individual was around one (3 to 16 falls per ten individuals a year [47,48]), these calculations (based on Chi-square test) were considered as a reasonable approximation for the confirmatory analysis using a negative-binomial regression model, which takes into account number of falls per individual.

Based on the trial by Spice et al. [21] we assumed an intra-cluster correlation coefficient of 0.02. Further, a cluster size of 10 was considered to be realistic. Therefore, about 40 clusters (382 individuals in all) have to be included in a balanced care allocation, to detect a clinically relevant reduction of one-year fall-risk from 33% to 20%, with 80% power at two-sided level of significance of 5%.

### Statistics

Analyses of the primary endpoint, number of falls per individual within a 12 months follow up period, will be performed on base of the full-analysis set (complete information regarding the primary outcome parameter). Within this population, all subjects are considered regardless to their actual compliance and the exercise-compliance of the responsible physiotherapists respectively (intention to treat principle). The statistical analysis of the primary endpoint will be considered at a two-sided level of significance of 5%.

For the confirmatory analysis of the primary endpoint, a negative binominal regression model [49] will be employed. This model approach takes repeated falls per subject into account and allows for further consideration of random effects as cluster (responsible physiotherapist) and region.

A detailed statistical analysis plan (SAP) will be defined in advance to the final analysis, to fix detailed statistical considerations with regard to further data analyses, multiple test issue, missing value imputation and sensitivity analyses.

## Discussion

Reducing falls in the elderly remains a major challenge. Falls are common in older persons and can cause notable decreases of quality of life due to fear of falling, restricted mobility and loss of autonomy as well as large costs for health care systems [1-3,50,51]. The available evidence suggests that currently available interventions to reduce falls have limited effectiveness and should target on risk population [2,16,17]. This could be due to the interventions themselves, however, it could also be that interventions were implemented and investigated in a suboptimal setting. Most fall prevention studies were planned in specialized settings. To reduce falls on a population-based level the question of the role of the general practitioners regarding the uptake of fall prevention programs by the "at risk person" still has to be further investigated. PreFalls focuses on the first health-system contact for elderly-their general practitioner. Already in the planning phase general practitioners were an important part of the interdisciplinary study team. The long-term relationship and the frequent contacts of general practitioners with their patients allow the efficient detection of persons who might be at high risk of falling. Trained staff members in the practices can support the physicians in using simple but well validated tests to reliably assess the risk. The multimodal intervention comprising group exercise in combination with an individualized home training program has been pre-tested successfully in another setting [40,41]. Local networks with trained physiotherapists or sport scientists have been set up to implement the intervention in a manner, which is both adequate and realistic for a primary care setting. From a methodological point of view it would have been preferable to randomize individual patients instead of practices, as this eliminates the possibility that recruitment processes in intervention and control practices differ. This has not been possible due to logistical reasons. While PreFalls is adequately powered to detect a clinically significant reduction in falls it is not powered to reliably detect potentially clinically relevant reduction of injurious falls, as the incidence of such events is comparably low. However, PreFalls follows current recommendations for fall prevention studies such as documenting both the number of persons falling and the number of falls per person. Careful measurement of quality of life and fear of falling is included, also monitoring of improvement in balance, body weight bearing, as well as motor coordination especially when performing activities of daily life.

We believe that with its strong focus on both a systematic and realistic fall prevention strategy adapted to a primary care setting PreFalls will be a valuable addition to the scientific literature in the field.

## Competing interests

The authors declare that they have no competing interests.

## Authors' contributions

MS, EF, PL and WB designed and managed the study. KL was responsible for criticizing the study design. Methodological support was yield by TS, KL and AS. WB, KL, MS and EF wrote the main part of the article. TS was responsible for defining the statistical approach and writing the statistical section of the manuscript. MH and KP reviewed the manuscript and gave valuable input for improvement. Grant application was organized by MS, EF, PL and MH. All of the authors have read and approved the final manuscript.

## Pre-publication history

The pre-publication history for this paper can be accessed here:

http://www.biomedcentral.com/1471-2318/11/7/prepub
